# Inflammatory processes involved in the alteration of liver function biomarkers in adult hospitalized patients treated with parenteral nutrition

**DOI:** 10.3389/fnut.2023.1034481

**Published:** 2023-01-27

**Authors:** Josep M. Llop Talaveron, Ana Suárez-Lledó Grande, Elisabet Leiva Badosa, Jordi Bas Minguet, Joan Climent Martí, Elisabet Poyatos Cantón, María B. Badia Tahull

**Affiliations:** ^1^Department of Pharmacy, Bellvitge University Hospital, Barcelona, Spain; ^2^Immunology Laboratory, Bellvitge University Hospital, University of Barcelona-IDIBELL, Barcelona, Spain; ^3^Immunology Laboratory, Germans Trias i Pujol Hospital, Barcelona, Spain

**Keywords:** parenteral nutrition, liver function biomarkers, inflammation, phytosterols, fish oil lipid emulsion

## Abstract

**Introduction:**

Liver damage has been associated with the accumulation of phytosterols (PS) in patients treated with parenteral nutrition (PN). We aimed to study the association of inflammatory markers with liver function biomarker (LFB) alterations in patients treated with PN containing PS.

**Materials and methods:**

Prospective observational study. Simple linear and stepwise multiple linear regression tests and interactions were performed.

**Results:**

Nineteen patients were included. In the multivariable model, determinations based on LFBs as dependent and phytosterols (and their fractions) as independent variables showed an association between increases in gamma-glutamyltransferase (GGT) and lanosterol (*p* < 0.001), stigmasterol (*p* < 0.001), interleukin-10 (IL-10) × total phytosterols (Phyt) (*p* < 0.009), tumor necrosis factor-α (TNF-α) × Phyt (*p* < 0.002), IL-10 × sitosterol (*p* < 0.002), TNF-α × sitosterol (*p* < 0.001), IL-10 × campesterol (*p* < 0.033), IL-10 (*p* < 0.006 and *p* < 0.015), TNF-α (*p* < 0.048 and *p* < 0.027). Increases in alanine aminotransferase (ALT) were associated with Phyt (*p* < 0.006), lanosterol (*p* < 0.016), C-reactive protein (CRP) × campesterol (*p* < 0.001), interleukin-6 (IL-6) × stigmasterol (*p* < 0.030), CRP (*p* < 0.08), and IL-6 (*p* < 0.042). Alkaline phosphatase (AP) increases were associated with CRP (*p* < 0.002).

**Discussion:**

Inflammation in the presence of plasmatic PS seems to have a synergistic effect in impairing liver function, mainly altering GGT but also ALT.

## 1. Introduction

Parenteral nutrition-associated liver disease (PNALD) is a common complication mainly occurring with long-term parenteral nutrition (PN) treatment and includes different pathologies, such as steatosis, cholestasis, fibrosis, cirrhosis, or liver failure. It is diagnosedvia its clinical presentation along with abnormal liver function biomarkers (LFBs): gamma-glutamyltransferase (GGT), alkaline phosphatase (AP), bilirubin (BI), and alanine aminotransferase (ALT) (1). The intravenous lipid emulsions (LEs) comprising PN are usually derived from vegetal sources and, therefore, contain natural phytosterols (PS), which are commonly associated with liver damage. This relationship has been described in the literature in both pediatric (2, 3) and adult patients (4, 5) treated with long-term PN, as well as in experimental studies (6–8).

Liver damage has been associated with the accumulation of PS; in practice, most protocols propose strategies to reduce the vegetal LE dose by replacing it with other LEs not containing PS, such as commercialized fish oil (FO) emulsions (9). One mechanism involved in liver damage is hepatic retention of PS, which has been suggested to interfere with the correct clearance of bile salts, leading to cholestasis (2, 5). Recently, the role of PS in hepatic alterations has been reinforced due to its proinflammatory activity in the liver (7).

In the acute critical patient, there are different risk factors such as surgery, cancer and infection-associated oxidative stress, which promote LFB elevation as well as the inflammatory response (1, 10, 11). In addition, in septic patients, liver injury can also be explained by the absorption of bacterial lipopolysaccharides (LPS) from the inflamed intestine (12).

No studies have been carried out to determine plasmatic PS values in hospitalized adults under PN treatment and, therefore, likely treated with only a few days of PN. This is probably because the clinical impact is considered low as LFBs tend to normalize after PN discontinuation. However, alterations with potential clinical impact can also be observed, especially in those patients on prolonged PN and critical patients presenting multiple organ failure (13, 14).

In a previous randomized clinical trial (RCT), we established that plasmatic PS accumulation and high GGT, AP and ALT values could be prevented with exclusive administration of FO LE (15). We compared patients treated with PN containing FO emulsion (without PS) or olive/soybean (O/S) LE. Subsequently, in a sub-study with the same population and design, we concluded that PS content varied among different LE brands and PS administered during PN resulted in accumulation (16).

In this sub-study, we have studied in the same group of patients whether the association between plasmatic PS and LFB alteration is conditioned by the presence of inflammation. The objective of this study is to determine whether the improvement of LFBs in patients treated with PN containing fish oil LE is due to their influence on inflammatory response mechanisms or to the absence of PS. For this purpose, we studied the association of inflammatory markers [C-reactive protein (CRP), tumor necrosis factor-α (TNF-α), and the cytokines IL-6 and IL-10], with LFB alterations in adult patients treated with PN containing PS.

## 2. Materials and methods

The present work was a sub-study of a previous RCT (15); here, in the same population, we analyzed different variables that were not evaluated in that prior study. This present sub-study was planned subsequent to the previous RCT (15).

### 2.1. Patients

The selected population corresponded to that included in the previous clinical trial, which studied the relationship between the type of LE used and liver function evolution *(EudraCT Number: 2014-003597-17*^1^) (15).

The population included in the initial study were patients that had received a minimum of 7 days of PN with a lipid intake of 0.8 g/kg/day of an O/S LE (established in our protocol), until they developed GGT alterations. They were then randomized 1:1 into LE groups: O/S or FO (omega-3 fatty acids, without PS) at a dose of 0.4 g/kg/day for a minimum of a further 7 days of PN.

### 2.2. Analytical parameters

Complementary to the main parameter determinations (LFBs and PS), the plasmatic values of inflammatory parameters were also determined at the beginning of the study (Day 0 or randomization day) and on Day 7 after randomization [*PS analysis was published in a separate paper* (15)]. Differences between the values of the aforementioned parameters on Days 7 and 0 were studied.

Quantification of cytokines:

– Sample collection: First, a clot was left to form for at least 30 min. Samples were then centrifuged at 700 × *g* within the first hour post-extraction and aliquoted and stored at −80°C until analysis (1 year later).
– Cytokine determination: serum values of IL-6, IL-10, and TNF-α were determined by Enzyme-Linked ImmunoSorbent Assay (ELISA), already commercialized and established for its use in the hospital laboratory. Errors due to manual handling were minimized by using a robot for processing the ELISA in a microplate.
– IL-6, IL-10, and TNF-α: each well of an ELISA multiplate for samples, standards and controls was coated with anti-lymphokine conjugated with biotin (for determining IL-6 or IL-10) or with anti-TNF-α antibodies conjugated with biotin (for determining TNF-α). After incubation and washing out, a streptavidin-HRP (horseradish peroxidase) solution was added to bind to the biotin present in each well. After the final incubation and washing-out, a chromogenic solution (HRP-H_2_O_2_-3,3′,5,5′-tetramethylbenzidine: TMB) was added to each well and catalyzed by HRP, changing its color. This final colored product, which was directly proportional to the cytokine concentration of the sample, was measured by absorbance.

### 2.3. Variables

Different variables were established to study the association of inflammatory markers with LFB alterations in adult patients treated with PN, and their relationship with the presence of plasmatic PS. The following variables were analyzed in the univariant and multivariant models:

– Dependent variables: variations in GGT, ALT, and AP (differences between initial and final values).
– Independent variables: final values of:
  – Total PS and different PS fractions, such as beta-sitosterol, campesterol, lanosterol, and stigmasterol.
  – Inflammatory markers: CRP, IL-6, IL-10, and TNF-α.

### 2.4. Statistical analysis

A descriptive statistical analysis was performed using frequency tables for all variables. For continuous variables, descriptive parameters such as sample size, median and interquartile range (IQR) were used; for categorical variables, percentages were given.

Simple linear regression tests and a stepwise multiple linear regression with interactions were performed in order to establish differences between LFB values according to the presence of PS and inflammatory parameters, and also for achieving a better adjustment. To avoid a co-linearity effect among the different inflammatory variables, the model was adjusted by residuals.

Statistical significance was established at *p* < 0.05. A multivariate approach was used to study the relationship between LFB variations and PS, and their fractions, CRP, TNF-α and interleukin values, and the interactions between these variables, thus a linear regression following a stepwise model with inclusion criteria of *p* < 0.2 was performed. Data were processed using the SPSS-IBM v22 statistical package.

## 3. Results

The study group included 19 patients (73.7% men, median age 68 years – IQR: 14.5 and 76 kg – IQR: 8.4). The median number of days of PN administration prior to inclusion in the study was eight (IQR 6) and, during this period, patients received an O/S LE at 0.8 g/kg/day. All patients had digestive system pathology, with 73.68% having cancer (cecum, colonic, gastric, gastrointestinal stromal, pancreatic, rectal neoplasia, and peritoneal carcinomatosis). Other digestive entities presented were adhesions (5.26%), mesenteric ischemia (5.26%), morbid obesity (5.26%), and occlusion (10.53%).

Table 1 displays baseline (Day 0 or randomization) and final values (Day 7) of the analytical parameters in each study arm. Statistically significant differences were detected for baseline CRP values (*p* = 0.04) between both groups and a tendency toward significance for baseline ALT values (*p* = 0.105).

**TABLE 1:** Analytical parameters: initial values (Day 0), final values (Day 7), and variation between these values.

Blood parameters	Initial value Day 0 Median (IQR)	Final value Day 7 Median (IQR)	Variation (differences) Median (IQR)
Gamma-glutamyltransferase (μkat/L)	3.14 (1.92)	2.93 (5.00)	−0.11 (2.04)
Alkaline phosphatase (μkat/L)	1.96 (0.99)	2.30 (1.49)	−0.16 (1.36)
Alanine aminotransferase (μkat/L)	0.43 (0.57)	0.29 (1.14)	−0.11 (0.37)
Bilirubin (μmol/L)	7.00 (3.50)	6.00 (4.50)	−1.00 (4.00)
Phytosterols (μg/ml)	21.30 (10.40)	19.80 (7.00)	−3.40 (4.85)
Sitosterol (μg/ml)	12.60 (4.95)	9.44 (7.52)	−3.76 (4.56)
Lanosterol (μg/ml)	1.08 (0.51)	0.53 (1.52)	−0.55 (0.27)
Campesterol (μg/ml)	2.14 (1.19)	1.94 (0.41)	−0.30 (0.74)
Stigmasterol (μg/ml)	0.74 (0.51)	0.54 (0.49)	−0.17 (0.47)
C reactive protein (mg/L)	99.1 (107.70)	60.80 (91.70)	−41.20 (63.95)
IL-6 (pg/ml)	39.00 (66.45)	30.80 (30.2)	−5.80 (25.25)
IL-10 (pg/ml)	1.06 (1.27)	1.58 (2.01)	0.00 (1.52)
Tumor necrosis factor-α (pg/ml)	17.25 (4.90	14.70 (5.6)	0.00 (2.31)

IQR, interquartile rank.

As we found in our RCT (15), on Day 7 after randomization, GGT, AP, beta-sitosterol, sitosterol, campesterol, lanosterol, and total PS significantly decreased in the FO group compared with the O/S group (Table 1).

When comparing each variable on Day 7 of treatment, statistically significant decreases in ALT were also found. TNF presented no significant changes, while in the FO group, CRP and IL-6 showed significant decreases and IL-10 showed a significant increase.

Among the other LFBs, bilirubin values were not considered as no significant variation was observed. Day 7 GGT, ALT, AP, IL-6, IL-10, CRP, and TNF-α values, as well as the differences calculated, did not follow a normal distribution. Therefore, a Napierian transformation was performed and a normal distribution was achieved; the Kolmogorov–Smirnov test presented a value of *p* = 0.200.

Table 2 depicts the simple linear regression performed to analyze the association of LFB variations with inflammatory parameters (measured as plasmatic cytokine concentration and CRP). The following statistically significant associations were observed: GGT values increased with high TNF-α values and AP increased with high CRP values. ALT values did not present any significant association with increases in the inflammatory parameters studied.

**TABLE 2:** Simple linear regression to analyze the association of liver function test variations and inflammatory parameters.

	Gamma-glutamyltransferase variation			Alkaline phosphatase variation			Alanine aminotransferase variation		
	***R* ^2^**	**B1 (CI 95%)**	***p***	***R* ^2^**	**B1 (CI 95%)**	***p***	***R* ^2^**	**B1 (CI 95%)**	***p***
CRP	0.069	0.116 (−0.102 to 0.334)	0.277	**0.227**	**0.212 (0.012 to 0.411)**	**0.039**	0.159	0.159 (−0.160 to 0.411)	0.309
IL-6	0.001	0.018 (−0.230 to 0.267)	0.877	0.000	0.006 (−0.224 to 0.256)	0.963	0.000	−0.002 (0.364 to 0.360)	0.993
IL-10	0.174	0.211 (−0.024 to 0.4469)	0.075	0.100	0.160 (−0.086 to 0.407)	0.188	0.033	0.134 (−0.236 to 0.504)	0.455
TNF-α	**0.269**	**0.740 (0.117 to 1.364)**	**0.023**	0.149	0.554 (−0.122 to 1.231)	0.102	0.076	0.571 (−0.450 to 1593)	0.254

CRP, C-reactive protein; TNF-α, tumor necrosis factor-α; *R*^2^, determination coefficient; B_1_, non-standard coefficient of regression slope; CI, confidence interval. Bold values represent the statistical significance.

Table 3 depicts the simple linear regression performed to analyze the association of LFB variation with plasmatic PS. Increases in GGT values were directly associated with statistically significant increases in total PS and certain fractions (sitosterol, lanosterol, and stigmasterol). Increases in AP did not associate with increases in PS or any PS fraction. Increases in ALT values were associated with increases in total PS and all their fractions studied, reaching statistical significance.

**TABLE 3:** Simple linear regression between variation in liver function biomarkers and total plasmatic phytosterols and their fractions 7 days after randomization and randomization day.

Variations in LFB parameters				
**Ln**	*R* ^2^ *	Ln total phytosterol (b1**)	**CI 95%**	**Sig.**
Gamma-glutamyltransferase	0.22	0.57	0.02 to 1.12	**0.043**
Alkaline phosphatase	0.04	0.26	−0.03 to 0.87	0.385
Alanine aminotransferase	0.29	0.96	0.20 to 1.73	**0.016**
Ln	*R* ^2^	Ln sitosterol	CI 95%	Sig.
Gamma-glutamyltransferase	0.27	0.47	0.07 to 0.87	**0.023**
Alkaline phosphatase	0.09	0.27	−0.18 to 0.72	0.218
Alanine aminotransferase	0.31	0.73	0.17 to 1.30	**0.014**
Ln	*R* ^2^	Ln campesterol	CI 95%	Sig.
Gamma-glutamyltransferase	0.16	0.47	−0.07 to 1.02	0.085
Alkaline phosphatase	0.01	0.14	−0.45 to 0.74	0.624
Alanine aminotransferase	0.22	0.79	−0.02 to 1.56	**0.044**
Ln	*R* ^2^	Ln lanosterol	CI 95%	Sig.
Gamma-glutamyltransferase	0.45	0.27	0.11 to 0.42	**0.002**
Alkaline phosphatase	0.14	0.15	−0.04 to 0.34	0.114
Alanine aminotransferase	0.30	0.31	0.07 to 0.56	**0.016**
Ln	*R* ^2^	Ln stigmasterol	CI 95%	Sig.
Gamma-glutamyltransferase	0.33	0.22	0.06 to 0.38	**0.010**
Alkaline phosphatase	0.04	0.08	−0.12 to 0.27	0.407
Alanine aminotransferase	0.237	0.27	0.02 to 0.53	**0.035**

*R^2^, determination coefficient.

**b1, non-standard coefficient of ln (phytosterols) of regression straight.

LFB, liver function biomarker; CI, confidence interval; Ln, Napierian logarithm. Bold values represent the statistical significance.

In the multiple linear regressions in stepwise analysis (Table 4), by adjusting the PS variables and their fractions with the cytokine and CRP variables, we found that the coefficient of determination *R*^2^ was higher than in the univariate model, suggesting association between inflammation and plasmatic PS. In these models, we observed that increases in AP were associated with CRP, independently of the presence of any PS fraction; however, the associations observed with GGT and ALT showed differences. Higher GGT values were associated with IL-10 coexisting with total PS and sitosterol and campesterol fractions, and with TNF-α in the presence of sitosterol. In addition, higher GGT values were associated with the presence of stigmasterol and lanosterol, independently of the inflammatory situation. On the other hand, high ALT values were associated with CRP in the presence of campesterol, and with IL-6 in the presence of stigmasterol, as well as with the presence of lanosterol, independently of the inflammatory load.

**TABLE 4:** Multiple linear regression. Stepwise: differences between liver parameter variations and risk factors (*p* < 0.2).

Variable	GGT variation		AP variation		ALT variation	
	***B* [95% CI]**	***p***	***B* [95% CI]**	***p***	***B* [95% CI]**	***p***
Total phytosterols					1.085 [0.358–1.813]	**0.006**
CRP			0.365 [0.152–0.577]	**0.002**		
IL-10 × total phytosterols	0.085 [0.024–0.145]	**0.009**			0.093 [−0.011 to 0.198]	0.076
TNF-α × total phytosterols	0.172 [0.073–0.271]	**0.002**				
***R*** ^2^	**0.577**		**0.435**		**0.424**	
CRP			0.365 [0.152–0.577]	**0.002**	0.137 [0.041–0.233]	**0.008**
IL-10 × sitosterol	0.121 [0.050–0.193]	**0.002**				
TNF-α × sitosterol	0.159 [0.077–0.240]	**0.001**				
***R*** ^2^	**0.641**		**0.435**		**0.347**	
Lanosterol	0.278 [0.155–0.400]	**0.000**			0.315 [0.067–0.563]	**0.016**
CRP			0.365 [0.152–0.577]	**0.002**		
IL-10	0.234 [0.078–0.390]	**0.006**				
***R* ^2^**	**0.662**		**0.435**		**0.298**	
IL-6					0.341 [0.013–0.669]	**0.042**
IL-6 × campesterol	0.095 [−0.018 to 0.209]	0.095				
CRP			0.365 [0.152–0.577]	**0.002**		
CRP × campesterol					0.360 [0.164–0.557]	**0.001**
IL-10 × campesterol	0.327 [0.030–0.625]	**0.033**				
TNF-α	0.541 [0.006–1.077]	**0.048**				
***R* ^2^**	**0.569**		**0.435**		**0.458**	
Stigmasterol	0.221 [0.100–0.342]	**0.001**				
IL-6 × stigmasterol					0.060 [0.006–0.113]	**0.030**
CRP			0.365 [0.152–0.577]	**0.002**		
IL-10	0.206 [0.047–0.365]	**0.015**				
TNF-α	0.520 [0.069–0.971]	**0.027**				
***R* ^2^**	**0.690**		**0.435**		**0.247**	

Variables are considered as Napierian logarithm.

CI, confidence interval; GGT, gamma-glutamyltransferase; AP, alkaline phosphatase; ALT, alanine aminotransferase; B, constant in the model *R*^2^ determination coefficient; CRP, C-reactive protein; IL-6, interleukin-6; IL-10, interleukin-10; TNF-α, tumor necrosis factor-α; Bold means statistically significant.

## 4. Discussion

Our results show that clinical situations associated with inflammation can induce LFB alterations; meanwhile, the plasmatic accumulation of PS can cause GGT and ALT alterations. But the most relevant result observed, with a consistent determination coefficient, is that inflammation in the presence of plasmatic PS seems to have a synergistic effect in impairing liver function, mainly altering GGT but also ALT, with no influence on AP. In this study, we found that the LFB alteration pattern of hospitalized adult patients on short-term PN treatment was different from that described in patients on long-term PN. In the present work, high GGT and AP values were found, whereas, in a previous study (5) performed in adult patients with Home Parenteral Nutrition (HPN) and, therefore, with elevated plasma PS concentrations, we found much higher ALT and BI values, with no differences in GGT and AP. This could be explained by the fact that increases in GGT and AP emerge as early indicators of hepatic dysfunction, while ALT and BI are linked to hepatocellular damage (11). BI is associated with intrahepatic cholestasis (17, 18) and 15 days of NP treatment are insufficient to appreciate any alteration in BI values. So, the differences observed in this study could be associated with the acute phase process of the pathology in hospitalized patients and the infectious and surgical aggressions that may occur during hospitalization.

Also, we found a positive association between plasmatic PS values (and their fractions) and GGT and ALT values, whereas this association was not seen for AP values (15). In another study in neonates with intestinal failure receiving long-term PN, Kurvinen et al. (19) also found that serum PS levels were positively associated with serum AST, ALT, and BI alterations, describing a different pattern of LFB alteration, but also a positive correlation with PS values.

It has been postulated that the hepatotoxic effect of PS takes place after long-term PN treatment (2, 4, 5, 9). However, this effect seems to also occur in patients receiving PN for shorter periods, such as critically ill patients, when inflammation is also present, and it appears to synergistically enhance the hepatotoxic effect of PS (1, 7). Furthermore, we observed different effects on LFBs based on the different PS fractions studied. Due to differences in the plasmatic clearance of PS, further complementary studies are required.

In this study, we assessed inflammation using a small series of parameters and considering different points of the inflammatory cascade. The stress injury response is characterized by an initial release of large amounts of proinflammatory cytokines IL-1 and TNF-α, which are primarily responsible for the production of other cytokines such as IL-6, the main acute phase cytokine (20–22). The proinflammatory cytokines TNF-α and IL-6 are produced from cells when the transcription factor NF-κB is activated in response to a variety of stimuli (23). The overregulation of TNF-α also stimulates the secretion of IL-6, which is the major inducer of acute phase reactants such as CRP (24), however, it has also anti-inflammatory properties, inhibiting the synthesis of TNF-α and IL-1β (25). The initial inflammatory response is controlled by immunomodulatory mechanisms; anti-inflammatory cytokines, such as IL-10, are involved in this immune response, limiting the potentially harmful effect of the inflammatory reaction. However, under pathological conditions, the anti-inflammatory response may be insufficient to counteract the inflammatory activity or it may be overcompensated and, thus, inhibit the immune system (25). Due to the complexity of analyzing inflammation parameters studying all cytokines involved in the inflammatory cascade and their facility of laboratory analysis, we decided to start analyzing the principal ones involved in activation and regulation of the cascade, as mentioned above: TNF-α, CRP, IL-6, and IL-10.

Recent experimental studies (7, 8) have evaluated the association between PS values, particularly stigmasterol, and the inflammatory response. There are some mechanisms described in these studies that could justify the association between inflammation and the presence of PS. Stigmasterol activation of Kupffer cells produces proinflammatory cytokines, which suppress nuclear receptor gene expression of transporters like the farnesoid X receptor (FXR), possibly contributing to cholestasis by suppressing the bile salt export pump (BSEP), thereby impairing the correct elimination of biliary salts (7). Other biological pathways under investigation include the activation of hepatic-based innate immunity mechanisms. Guthrie et al. (8) evidenced this synergy in an experimental study where they aimed to elucidate whether PS caused inflammation in hepatocytes or Kupffer cells independently or required co-stimulation by LPS. They concluded that PS alone do not cause the inflammatory response, but rather synergistically maximize the inflammatory response in Kupffer cells previously activated by LPS. Furthermore, the study of El Kasmi et al. (7) suggested that stigmasterol may enhance the inflammatory response in Kupffer cells previously activated by exposure to inflammatory mediators from bacterial translocation or sepsis. In both studies (7, 8), the PS fraction studied was stigmasterol, which is a compound of PN emulsions with a slow clearance, as we confirmed in our previous study (15), thereby conferring greater exposure to them.

As Guthrie et al. (8) postulated that PS alone were not significantly associated with liver inflammation, but that this could occur in states associated with sepsis, this association between PS and other clinical situations related to the inflammatory response cannot be ruled out. Medical situations, such as those occurring after surgery, could generate an immune response that could also interact at the cytokine level with Toll-like receptors (TLR4 type) (26) and, therefore, also act synergistically with plasmatic PS. Within such a framework, it is important to consider that, following surgery, excessive innate immune responses or failed adaptive immune responses can result in significant morbidity and mortality from systemic response syndrome, infection and sepsis.

As described by Alazawi et al. (27) tissue injury and infection are sensed by a group of protein receptors that can be activated by pathogen-associated molecular patterns and damage-associated molecular patterns, also known as alarmins, which are host molecules liberated as a result of tissue destruction. Membrane-spanning Toll-like receptors (26, 27) have been included among these molecular patterns described. The authors (27) finally concluded that local immune responses to surgery led to systemic pro-inflammatory and immunosuppressive phases that were temporally related and proportionate in magnitude.

Therefore, a better comprehension of these mechanisms could have implications for clinical study designs and should lead to the emergence of new research related to the presence of PS in patients treated with PN.

## 5. Conclusion

The interaction and possible synergy between the PS administered in PN and inflammatory mediators allow for new comprehensive approaches to PNALD: LFB elevation might be associated not only with the presence of PS but also with a hepatic inflammatory response that could add synergic effect.

Thus, a possible new strategy in PN treatment could be the use of FO LE, where possible; additional benefits would be the absence of PS and their anti-inflammatory activity.

This is a first approach that should be complemented with studies including a larger number of patients and more inflammatory markers.

## 6. Limitations

Considering the limitations of this study, it is important to highlight that this was not a primary study. Another important limitation is that this study included a small cohort of patients, with a small number of patients studied.

Another limitation was the need to perform a Napierian transformation to achieve a normal distribution in the initial results obtained.

It is also relevant to note that, in the previous RCT (15), the inflammatory state was not considered during randomization. In fact, there were significant differences in baseline CRP values between both arms of the study. For this reason, and given that we also previously demonstrated (16) that the value of phytosterols and their fractions was significantly associated with the type of emulsion used and with LFB alterations, to study the impact of the inflammatory state on LFB, we designed the statistical approximation for a single sample that grouped both arms adjusted for the plasmatic PS value.

The sub-study did not address the direct impact of FO emulsions (without PS) on inflammation indicators, but rather the relevance of inflammatory processes in LFB alterations.

Furthermore, in this study, we did not consider all cytokines involved in the inflammatory cascade, but we have shown an association between inflammation and alteration in LFBs.

Finally, we did not study other factors associated with PNALD in critical patients, although they usually require support with PN, because they were excluded from this study.

## 7. Clinical relevancy statement

This study sheds light on other factors involved in the liver damage of patients treated with PN, showing that clinical situations associated with inflammation can induce alteration of LFB; meanwhile, the plasmatic accumulation of PS alone can cause transaminase alteration, as previously demonstrated.

## Data availability statement

The raw data supporting the conclusions of this article will be made available by the authors, without undue reservation.

## Ethics statement

The studies involving human participants were reviewed and approved by the Ethical Committee of Bellvitge University Hospital. The patients/participants provided their written informed consent to participate in this study.

## Author contributions

MB, JL, EL, and AS-L contributed to the conception and design of the research and writing of the manuscript. JB, JC, and EP contributed to the final review of this manuscript. All authors critically revised the manuscript, agreed to be fully accountable for ensuring the integrity and accuracy of the work, read, and approved the final manuscript.

## Footnotes

1
www.clinicaltrialsregister.eu

## References

[1] Zaloga G. Phytosterols, lipid administration, and liver disease during parenteral nutrition. *JPEN J Parenter Enteral Nutr.* (2015) 39(1 Suppl.):39S–60S. 10.1177/0148607115595978 26177665

[2] Clayton P Whitfield P Iyer K. The role of phytosterols in the pathogenesis of liver complications of pediatric parenteral nutrition. *Nutrition.* (1998) 14:158–64. 10.1016/S0899-9007(97)00233-5 9437703

[3] Gura K Duggan C Collier S Jennings R Folkman J Bistrian B Reversal of parenteral nutrition-associated liver disease in two infants with short bowel syndrome using parenteral fish oil: implications for future management. *Pediatrics.* (2006) 118:E197–201. 10.1542/peds.2005-2662 16818533

[4] Ellegård L Sunesson A Bosaeus I. High serum phytosterol levels in short bowel patients on parenteral nutrition support. *Clin Nutr.* (2005) 24:415–20. 10.1016/j.clnu.2005.01.001 15896428

[5] Llop J Virgili N Moreno-Villares J García-Peris P Serrano T Forga M Phytosterolemia in parenteral nutrition patients: implications for liver disease development. *Nutrition.* (2008) 24:1145–52. 10.1016/j.nut.2008.06.017 18656327

[6] Carter B Taylor O Prendergast D Zimmerman T Von Furstenberg R Moore D Stigmasterol, a soy lipid-derived phytosterol, is an antagonist of the bile acid nuclear receptor FXR. *Pediatr Res.* (2007) 62:301–6. 10.1203/PDR.0b013e3181256492 17622954

[7] El Kasmi K Anderson A Devereaux M Vue P Zhang W Setchell K Phytosterols promote liver injury and Kupffer cell activation in parenteral nutrition-associated liver disease. *Sci Transl Med.* (2013) 5:206ra137. 10.1126/scitranslmed.3006898 24107776 PMC4070735

[8] Guthrie G Tackett B Stoll B Martin C Olutoye O Burrin DG. Phytosterols synergize with endotoxin to augment inflammation in Kupffer cells but alone have limited direct effect on hepatocytes. *JPEN J Parenter Enteral Nutr.* (2018) 42:37–48. 10.1177/0148607117722752 28792854 PMC8279067

[9] Nandinava P Carlson S Chang M Cowan E Gura K Puder M. Treatment of parenteral nutrition-associated liver disease: the role of lipid emulsions. *Adv Nutr.* (2013) 4:711–7. 10.3945/an.113.004770 24228202 PMC3823519

[10] Moreno Villares J. Complicaciones hepáticas asociadas al uso de nutrición parenteral. *Nutr Hosp.* (2008) 23(Suppl. 2):25–33. 18714408

[11] Llop-Talaveron J Badia-Tahull M Lozano-Andreu T Suarez-Lledo A Leiva-Badosa E. Risk factors of hepatic function alterations in hospitalized adult patients treated with short-term parenteral nutrition receiving the same lipid composition at the same dose. *Lipids Health Dis.* (2018) 17:267. 10.1186/s12944-018-0912-4 30474548 PMC6260870

[12] Lee W Sokol R. Intestinal microbiota, lipids and the pathogenesis of the intestinal failure-associated liver disease. *J Pediatr.* (2015) 167:519–26. 10.1016/j.jpeds.2015.05.048 26130113 PMC4554799

[13] Vaquerizo Alonso C Mesejo A Acosta Escribano J Ruiz Santana S. Management of parenteral nutrition in intensive care units in Spain. *Nutr Hosp.* (2013) 28:1498–507. 24160207 10.3305/nh.2013.28.5.6815

[14] Grau T Bonet A Rubio M Mateo D Farré M Acosta J Liver dysfunction associated with artificial nutrition in critically ill patients. *Crit Care.* (2007) 11:R10. 10.1186/cc5670 17254321 PMC2147066

[15] Llop Talaveron J Badía-Tahull M Lozano-Andreu T Rigo-Bonin R Virgili-Casas N Farran –Teixidó L Phytosterolemia and gamma-glutamyltransferase in adults with parenteral nutrition: fish versus vegetal lipids, randomized clinical trial. *Nutrition*. (2020) 70:110587. 10.1016/j.nut.2019.110587 31743812

[16] Llop-Talaveron J Leiva-Badosa E Novak A Rigo-Bonnin R Ticó-Grau J Suñé-Negre J Phytosterolaemia associated with parenteral nutrition administration in adult patients. *Br J Nutr.* (2020) 123:1365–72. 10.1017/S0007114520000574 32077392

[17] Vanwijngaerden Y Langouche L Brunner R Debaveye Y Gielen M Casaer M Withholding parenteral nutrition during critical illness increases plasma bilirubin but lowers the incidence of biliary sludge. *Hepatology.* (2014) 60:202–10. 10.1002/hep.26928 24213952

[18] Siddique A Kowdley K. Approach to a patient with elevated serum alkaline phosphatase. *Clin Liver Dis.* (2012) 16:199–229. 10.1016/j.cld.2012.03.012 22541695 PMC3341633

[19] Kurvinen A Nissinen M Andersson S Korhonen P Ruuska T Taimisto M Parenteral plant sterols and intestinal failure-associated liver disease in neonates. *J Pediatr Gastroenterol Nutr.* (2012) 54:803–11. 10.1097/MPG.0b013e3182474118 22197940

[20] Mayer J Rau B Gansauge F Beger H. Inflammatory mediators in human acute pancreatitis: clinical and pathophysiological implications. *Gut.* (2000) 47:546–52. 10.1136/gut.47.4.546 10986216 PMC1728074

[21] Calder P. Marine omega-3 fatty acids and inflammatory processes: effects, mechanisms and clinical relevance. *Biochim Biophys Acta.* (2015) 1851:469–84. 10.1016/j.bbalip.2014.08.010 25149823

[22] Serhan C. Pro-resolving lipid mediators are leads for resolution physiology. *Nature.* (2014) 510:92–101. 10.1038/nature13479 24899309 PMC4263681

[23] Hayden MS Ghosh S. Regulation of NF-κB by TNF family cytokines. *Semin Immunol.* (2014) 26:253–66. 10.1016/j.smim.2014.05.004 24958609 PMC4156877

[24] Heinrich P Castell J Andus T. Interleukin-6 and the acute phase response. *Biochem J.* (1990) 265:621–36. 10.1042/bj2650621 1689567 PMC1133681

[25] De Pablo Sanchez R Montserrat Sanz J Prieto Martin A Reyes Martin E Álvarez de Mon Soto M Sánchez García M. Balance entre citocinas pro y antiinflmatorias en estados sépticos. *Med Intensiva.* (2005) 29:151–8. 10.1016/S0210-5691(05)74222-4

[26] Akira S Takeda K. Toll-like receptor signalling. *Nat Rev Immunol.* (2004) 4:499–511. 10.1038/nri1391 15229469

[27] Alazawi W Pirmadjid N Lahiri R Bhattacharya S. Inflammatory and immune responses to surgery and their clinical impact. *Ann Surg.* (2016) 264:73–80. 10.1097/SLA.0000000000001691 27275778

